# Knowledge about Cervical Cancer and Barriers of Screening Program among Women in Wufeng County, a High-Incidence Region of Cervical Cancer in China

**DOI:** 10.1371/journal.pone.0067005

**Published:** 2013-07-02

**Authors:** Yao Jia, Shuang Li, Ru Yang, Hang Zhou, Qunying Xiang, Ting Hu, Qinghua Zhang, Zhilan Chen, Ding Ma, Ling Feng

**Affiliations:** 1 Department of Gynecology and Obstetrics, Tongji Hospital, Tongji Medical College, Huazhong University of Science and Technology, Wuhan, P.R. China; 2 Maternity and Child Health Care Hospital, Tujia Nationality Autonomous County, Wufeng, P.R. China; Kinghorn Cancer Centre, Garvan Institute of Medical Research, Australia

## Abstract

**Purpose:**

Cervical cancer screening is an effective method for reducing the incidence and mortality of cervical cancer, but the screening attendance rate in developing countries is far from satisfactory, especially in rural areas. Wufeng is a region of high cervical cancer incidence in China. This study aimed to investigate the issues that concern cervical cancer and screening and the factors that affect women’s willingness to undergo cervical cancer screening in the Wufeng area.

**Participants and Methods:**

A cross-sectional survey of women was conducted to determine their knowledge about cervical cancer and screening, demographic characteristics and the barriers to screening.

**Results:**

Women who were willing to undergo screenings had higher knowledge levels. “Anxious feeling once the disease was diagnosed” (47.6%), “No symptoms/discomfort” (34.1%) and “Do not know the benefits of cervical cancer screening” (13.4%) were the top three reasons for refusing cervical cancer screening. Women who were younger than 45 years old or who had lower incomes, positive family histories of cancer, secondary or higher levels of education, higher levels of knowledge and fewer barriers to screening were more willing to participate in cervical cancer screenings than women without these characteristics.

**Conclusion:**

Efforts are needed to increase women’s knowledge about cervical cancer, especially the screening methods, and to improve their perceptions of the screening process for early detection to reduce cervical cancer incidence and mortality rates.

## Introduction

Worldwide, cervical cancer is the second most common cause of cancer-related deaths [1] and is responsible for approximately 250,000 annual deaths [2], most of which occur in developing countries [3]. The incidence of cervical cancer has been controlled in developed countries due to the widespread use of cervical cancer screening systems, especially the systemic use of the Papanicolaou (Pap) smear [4], [5]. It is notable that globally, developing countries spend only 5% of their resources on cancer, thus leading to higher incidence and mortality rates in these counties.

Like many other developing countries, China faces a heavy burden with regard to women's health. China accounts for 29% of the 51,000,000 new cases of cervical cancer each year [6]. Cervical cancer ranks as the eighth most common malignant cancer among females in Mainland China, with an age-standardized incidence rate (ASIR) of 5.15 per 100,000 inhabitants. Although the incidence of cervical cancer in China is low in comparison to that of western countries, the mortality rate remains high, especially in rural areas [7].

In many developing countries, cervical cancer screenings are unavailable or are poorly accessible. Although China has provided free cervical screenings in some rural areas for women 35 to 59 years of age since 2009 [8], the public still has limited knowledge about cervical cancer, and thus, women are less willing to undergo screenings. Educational levels and misconceptions might also contribute to the low screening attendance [9].

Wufeng is a typical low-income county located in central China, with a total population of approximately 208,000, which includes 96,000 women. More than 80% of the local residents are of the Tu Minority. This is a high-incidence region for cervical cancer in China. In the early 1970s, the standardized prevalence rate of cervical cancer was 1320.01/100,000, and the standardized mortality rate was 66.03/100,000, which was the highest mortality rate in China [10], [11]. From 2004 to 2005, 188 women in this county died of malignant tumors (mortality rate: 0.96‰), of which 46 were from cervical cancer (mortality rate: 0.23‰) [12].

In the Wufeng area, health care services are provided by only 10 health centers that cover a population of 0.21 million. Pap smears, visual inspections with acetic acid, and colposcopy are currently used at the public health clinics in the Wufeng area. This situation demonstrates how the success of cervical cancer screening programs depends on the achievement of proper coverage to reduce the cervical cancer morbidity and mortality rates. However, improvements in cervical cancer screenings are insufficient to increase screening attendance; thus, the factors that influence women’s willingness to undergo screenings must be understood. The purpose of this study was to explore the knowledge regarding cervical cancer and the potential barriers to screening in the local population.

## Materials and Methods

### Design and Sample

This study was approved by the Ethics Committee of Tongji hospital, Tongji Medical College, Huazhong University of Science and Technology, PR China (http://clinicaltrials.gov; NCT01267851). All participants provide their written consent to participate in this study.

The study employed a cross-sectional design and used a nonprobability sampling method known as convenience sampling [9]. A total of 7929 local women were recruited in our investigation. A total of 5936 women over 26 years of age, who were sexually active and willing to participate in the survey, were recruited between 2006 and 2008. Ultimately, 5929 questionnaires (99.9%) were determined to be valid. All participants possessed local household identity from three towns in Wufeng County (Caihua Town, Fujiayan Town and Changleping Town). Door-to-door recruiting for the investigation and free-of-charge gynecological examinations were performed by staff to attract participants to the centers. Face-to-face interviews were conducted in person by professionally trained investigators. The completed questionnaires were returned directly to the researchers. After the face-to-face interviews, all participants were invited to undergo a free gynecological examination, including cervical cancer screening (visual inspection with acetic acid, visual inspection with Lugol’s iodine, colposcopy and biopsy et al). We did not provide detailed knowledge or educational materials about cervical cancer and gynecological examinations before the investigation.

### Measures

The questionnaire consisted of 4 parts: demographic information, knowledge about cervical cancer, willingness to participate in cervical cancer screening programs and potential barriers to attending screenings.

The section on cervical cancer knowledge consisted of 16 items, including 7 general cervical cancer knowledge items, 4 items about the cervical cancer screening method and 5 items about early-stage treatments for cervical cancer. Each correct answer was given 1 point, and the total possible score for this section ranged from 0 to 16.

Cervical cancer is a sensitive topic in the Wufeng area. To indirectly assess their willingness to undergo cervical cancer screening, the participants were required to answer the question “Would you like your relatives attend a cervical cancer screening?” We used the indirect question strategy to reduce the influences of the sensitive nature of the topic and the gynecological examination on the willingness of participants to undergo cervical cancer screening [13], [14].

The section on potential barriers to attending screening contained 8 options: (1) No symptoms/discomfort; (2) Anxious feeling once the disease was diagnosed; (3) Afraid to be deceived; (4) Afraid of pain during screening; (5) Cervical cancer is incurable even if screening is effective; (6) Do not know the benefit of cervical cancer screening; (7) Husband disapproves of cervical cancer screening; and (8) Other: details needed. The participants could choose more than one option.

Although no formal validation of this questionnaire was performed, we performed quality controls, interviewer training, and extensive reviews throughout the entire study.

### Data Analysis

The questionnaire answers were recorded twice and verified three times. After logic and outlier verification, the rates of logic and outlier errors were ensured to be less than 0.2%. We used a χ2 test to examine the differences in demographic variables between women with different levels of willingness to undergo cervical cancer screening. Student’s *t* test was used to determine whether there were differences in the knowledge scores between the groups with different levels of willingness. Logistic regression was used to indicate the demographic factors, the cervical cancer knowledge levels, and the barriers to screening. The factors included in the logistic regression model were income, age, education, family history, cervical cancer knowledge level and barriers to screening. All statistical analyses were performed with SPSS 13.0, and statistical significance was defined as P<0.05.

## Results

### Demographic Characteristics of Women with Different Levels of Willingness to Undergo Cervical Cancer Screening

A total of 5929 valid questionnaires were collected. The ages of women in the study ranged from 26 to 65 years. As shown in Table 1, 1692 women (28.5%) had at least a secondary education level, the majority (72.4%) had yearly incomes of more than 560 US dollars, and 660 had family histories of cancer. A total of 4149 women expressed their willingness to participate in cervical cancer screenings. A significant difference was identified in age relative to women of different levels of willingness to undergo screenings (P<0.01) such that women older than 45 years were more willing to undergo cervical cancer screenings.

**Table 1:** Demographic characteristics of women of different willingness towards cervical cancer screening (n = 5929) (From χ^2^ test).

Demographic characteristic	Frequency of women willing to participate in screening	Frequency of women not willing to participate in screening	Total frequency	*P* value
Age(years)				0.00
≤45	2531(42.7%)	977(16.5%)	3508(59.2%)	
>45	1618(27.3%)	803(13.5%)	2421(40.8%)	
Education status				0.00
Primary or below	2872(48.4%)	1365(23.0%)	4237(71.5%)	
Secondary or above	1277(21.5%)	415(7.0%)	1692(28.5%)	
Income (US dollars per year)				0.68
≤560	1150(19.4%)	484(8.2%)	1634(27.6%)	
>560	2999(50.6%)	1296(21.9%)	4295(72.4%)	
Family history of cancer				0.00
Positive	507(8.6%)	153(2.6%)	660(11.1%)	
Negative	3642(61.4%)	1642(27.4%)	5269(88.9%)	

A significant difference was found in the educational levels of the participants with different levels of willingness (P<0.01); 75.5% of women who had achieved a secondary or higher education level were willing to undergo screenings, compared with 68.5% of women with lower educational levels. Women with positive family histories of cancer were more willing to participate in screening activities than those with negative histories (P<0.01).

### Women’s Cervical Cancer Knowledge Levels

The mean total knowledge level score for all participants was 6.91 (SD, 3.42) out of a possible range from 0 to 16. There was a significant difference in the total knowledge levels between women with different levels of willingness (P<0.01) such that women who were willing to undergo screenings had higher knowledge levels. Women with higher education and income levels had higher levels of knowledge (P<0.01). Women with positive family histories of cancer were more likely to have higher levels of knowledge about cervical cancer (P<0.01).

### Common Knowledge about Cervical Cancer

The mean knowledge score for the cervical screening items for all respondents was 4.27 (SD, 1.50) out of a possible range of 0 to 7, and significant differences were observed between women of different ages, educational levels, incomes, and family histories of cancer (Table 2). A significant difference was identified with regard to knowledge about cervical cancer screenings between the women who were willing to undergo screenings (mean, 4.71 [SD, 1.22]) and those who were unwilling (mean, 3.23 [SD, 1.57]; P<0.01). As shown in Table 3, the score for the 7 individual items was also significant, with screened women more likely to answer these items correctly.

**Table 2:** Factors influencing cervical cancer knowledge levels.

Influencing factors	Score	*P* value
Age		0.00
≤45	7.05±3.45	
>45	6.72±3.36	
Education status		0.00
Primary or below	6.74±3.43	
Secondary or above	7.34±3.37	
Income(US dollars per year)		0.00
≤560	6.48±3.50	
>560	7.08±3.38	
Family history of cancer		0.00
Positive	7.53±3.33	
Negative	6.84±3.42	
Willingness to participate in screening		0.00
Yes	7.63±3.05	
No	5.24±3.66	

**Table 3:** Frequency of correct answer for items about cervical cancer (n = 5929) (From χ^2^ test).

Item		Frequency of correct answer among women willing to participate in screening		Frequency of correct answer among womennot willing to participate in screening		Total frequency		*P* value
Part 1. Common knowledge about cervical cancer	There is early stage of cervical cancer.							
			2018(48.60%)		389(21.9%)		2407(40.6%)	0.00
	Cervical cancer is curable if detected early.							
			3616(87.2%)		1175(66.0%)		4791(80.8%)	0.00
	HPV infection is a necessary factor inducing cervical cancer.							
			167(4.0%)		9(0.5%)		176(3.0%)	0.00
	HPV could transmit sexually.							
			2594(62.5%)		575(32.3%)		3169(53.4%)	0.00
	Having multiple sex partners is a risk factor of cervical cancer.							
			3698(89.1%)		1085(61.0%)		4783(80.7%)	0.00
	Maintaining sexual hygiene could prevent cervical cancer.							
			4019(96.9%)		1334(74.9%)		5353(90.3%)	0.00
	Fruit, vegetables could prevent cervical cancer.							
			3438(82.9%)		1186(66.6%)		4624(78.0%)	0.00
	
Part 2. Screening method for cervical cancer	Pap smear		689(16.6%)		215(12.1%)		904(15.2%)	0.00
	Visual inspection with acetic acid		525(12.7%)		203(11.4%)		728(12.3%)	0.18
	Colposcopy		645(15.5%)		237(13.3%)		882(14.9%)	0.03
	HPV test		459(11.1%)		196(11.0%)		655(11.0%)	0.95
	
Part 3. Treatment of early stage cervical cancer	Electric cauterization		2856(68.8%)		790(44.4%)		3646(61.5%)	0.00
	Surgery		3240(78.1%)		988(55.5%)		4228(71.3%)	0.00
	Cryosurgery		752(18.1%)		267(15.0%)		1019(17.2%)	0.00
	Laser		1360(32.8%)		428(24.0%)		1788(30.2%)	0.00
	Conization		1584(38.2%)		251(14.1%)		1835(30.9%)	0.00

The participants, especially the women willing to participate in screening, had a high cognitive level with regard to common knowledge about cervical cancer. Most of them recognized that cervical cancer was curable if detected early (80.8%), and most knew that it could be prevented by having fewer sexual partners (80.7%), maintaining sexual hygiene (90.3%), and eating more fruits and vegetables (78.0%). Only 40.6% of the respondents knew the early stage of cervical cancer (cervical intraepithelial neoplasia). Many of the participants knew that HPV could be transmitted sexually (53.4%), but the relationship between HPV infection and cervical cancer was poorly understood (3.0%).

### Screening Method for Cervical Cancer

The mean knowledge score regarding cervical cancer screening methods was 0.53 (SD, 1.27), out of a possible range of 0 to 4, for all responders. A significant difference was identified in the levels of knowledge about cervical cancer screening methods between women of different levels of willingness (P = 0.01). As shown in Table 3, women who were willing to participate in cervical cancer screenings were more aware of the common “Pap smear” and “colposcopy” methods for cervical cancer screening (P<0.05).

### Treatment of Early-stage Cervical Cancer

The mean knowledge score regarding early-stage treatments was 2.11 (SD, 1.69), out of a possible range of 0 to 5, for all participants. There was also a significant difference between women of different levels of willingness to undergo screening, and women who were willing to participate in screenings were more likely to correctly answer the questions related to the “electric cauterization”, “surgery”, “cryosurgery”, “laser” and “conization” treatment methods (P<0.05).

### Barriers to Participating in Cervical Cancer Screening

The frequencies of the reported barriers were analyzed, and the results are shown in Table 4. “Anxious feeling once the disease was diagnosed” (47.6%), “No symptoms/discomfort” (34.1%) and “Do not know the benefit of cervical cancer screening” (13.4%) were the top three reasons for refusing cervical cancer screening.

**Table 4:** Barriers towards participate in cervical cancer screening.

Barriers	Frequency
Anxious feeling once the disease was diagnosed	2825(47.6%)
No symptoms/discomfort	2023(34.1%)
Do not know the benefit in cervical cancer screening	795(13.4%)
Afraid to be deceived	734(12.4%)
Afraid of pain during screening	673(11.4%)
Cervical cancer is incurable even if screening is effective	660(11.1%)
Husband disapproves of cervical cancer screening	130(2.2%)

### Factors Predictive of Screening Willingness

Table 5 shows the odds ratios with logistic regression for the predictors of willingness to attend a cervical cancer screening. The significant factors identified were age, education status, income, family history of cancer, total knowledge score regarding cervical cancer and the number of barriers to cervical cancer screening. Younger participants (≤45 years), those in the low-income group, those who had a secondary or higher level of education, those who had positive family histories of cancer, those with a better knowledge of cervical cancer and those who perceived fewer barriers to screening were more likely to attend cervical cancer screenings.

**Table 5:** Factors predicted by demographic characteristics, cervical cancer knowledge levels as well as barriers towards cervical cancer screening.

Variables	*P* value	Exp(B)	95% CI
Age (>45years vs. ≤45 years)	0.01	0.86	0.76–0.97
Education state (Secondary or above vs. Primary or below)	0.00	1.29	1.12–1.49
Income (>560 vs. ≤560 US dollars per year)	0.00	0.81	0.71–0.93
Family history of cancer (Positive vs. Negative)	0.01	1.29	1.06–1.58
Cervical cancer knowledge levels	0.00	1.28	1.25–1.31
Number of barriers towards cervical cancer screening	0.00	0.91	0.88–0.95
Constant	0.00	0.61	

## Discussion

### Factors Affecting the Willingness to Undergo Cervical Cancer Screening

Numerous studies have emphasized the knowledge levels and possible factors that affect screening behaviors among women in different countries around the world. The results have shown that not only demographic characteristics but also knowledge about cervical cancer can affect cervical cancer screening participation [15], [16]. To date, however, subject willingness as an intrinsic and predictive factor of individual behavior has received very little attention [17]. One rather surprising and important finding from this study is that the majority of surveyed women (70%) indicated that they were willing to participate in cervical cancer screenings. Women who were willing to undergo screenings had a higher level of cervical cancer knowledge than women who were unwilling. However, in both groups, most of the women did not know that “HPV infection is a necessary factor for cervical cancer induction”, and most had little knowledge of screening methods and treatments. We perceived it necessary to popularize cervical cancer knowledge in the Wufeng area.

Many of the demographic variables that were found to correlate with cervical cancer screening behaviors in previous studies were also found to correlate with screening willingness in our study [18]–[20].

Women who were willing to undergo screenings tended to be younger and better educated and to have higher incomes. In our study, similar results were found with the χ2 test, and a logistic regression showed that women who younger than 45 years of age were more willing to undergo cervical cancer screening, even if they had lower incomes, which contradicted our expectations.

The most commonly reported barrier that affected the participants’ willingness to attend screenings was the anticipated feeling of anxiety if the screening revealed an illness, which indicates that anxiety may be a negative motivator of screening attendance. This result is consistent with a previous review by Ackerson [21], who suggested that the anxiety is due to a lack of information about the intent of cervical cancer screenings. Another important barrier cited by the participants was that of “no symptoms/no discomfort”, which indicates that signs and symptoms related to cervical cancer might encourage screening behaviors. The focus on health crises rather than disease prevention is an element of Chinese culture. For the Chinese, as with most Asians, visits to the physician do not occur unless there are obvious abnormalities. This finding is also supported by previous studies in which Chinese women appeared to participate in health care initiatives for reasons of illness rather than prevention [22], [23]. Other barriers included not knowing the benefits of cervical cancer screenings, the fear of deception, the fear of pain during screening, and the possibility of incurable cervical cancer even if the screening is effective. These barriers draw our attention to the need for extensive public education intended to correct these misconceptions, with an emphasis on the fact that cervical cancer screenings can detect precancerous lesions that occur early in the course of the cancer and can thus be mitigated by certain treatments. Unexpectedly, a few participants declared that their husband’s disapproval of screening was a barrier, a finding that is inconsistent with other studies [24], [25].

### The Effect of Knowledge about Cervical Cancer on the Level of Willingness to Undergo Cervical Cancer Screenings

The knowledge level about cervical cancer was low among the participants, although women who were willing to participate in the screenings had a higher knowledge level. Studies of the various areas of knowledge have demonstrated that the lack of knowledge about cervical cancer appears to be an important barrier to participation in cervical cancer screenings [15], [16], [26].

The majority of participants correctly recognized that having multiple sex partners is a cervical cancer risk factor. Protective actions, such as the consumption of fruits and vegetables and the maintenance of sexual hygiene, were identified by the majority of the women. However, the awareness of the role of HPV in cervical cancer etiology was low, which is consistent with previous studies [15], [27]. Therefore, it is not surprising that few of the women knew that the use of the HPV DNA test as a cervical cancer screening and management tool was expanding. The participants’ knowledge level of screening methods and treatments, two important elements of knowledge about cervical cancer, was very low, thereby indicating an incomplete understanding of the subject.

Our findings also supported the health belief model; the women’s knowledge of cervical cancer was shown to correlate with participation in cervical cancer screenings. Although a national cervical cancer-screening program has been promoted in different areas in China, the lack of knowledge about cervical cancer remains an important factor that affects the participation of women in these screening programs. Therefore, accurate information about cervical cancer must be made available so that women can acquire knowledge about cervical cancer, especially the risk factors, screening methods, treatments and importance of regular screenings for early diagnosis. Health providers, who represent a major source of health education, have been shown to greatly influence women’s screening behaviors [28], which suggests that community health workers and other health providers are important components of any program that seeks to increase cervical cancer screening rates.

### Limitations

The participants were recruited by convenience sampling, a nonprobability sampling method that inevitably limits the results. The women who participated in the survey were recruited from the rural areas of Wufeng and were characterized by low incomes and low educational levels. Therefore, the results should not be generalized to all women in China, particularly those in urban areas. While the sample size was very large, it was a convenience sample of women who attended the interviews to obtain a free gynecological examination. Participants received a free-of-charge gynecological examination that included cervical cancer screening after the interview. These two factors might affect the statistical result of willingness to undergo cervical cancer screening. The interviews were performed at three centers, and the inconvenience caused by traffic might have interfered with participation. Additionally, this study had a cross-sectional design that prevented us from inferring the actual relationship between the women’s screening behaviors and their knowledge of cervical cancer. A longitudinal study is therefore warranted to fully examine the impact of knowledge on participant behaviors. Despite these limitations, the results of this study provide a basis for future study.

### Conclusions

This is the first study to provide insight into the factors that affect women’s participation in cervical cancer screenings in rural areas of China. The study showed that women in rural areas lacked knowledge about cervical cancer and the importance of cervical screening for early cancer diagnosis. Cervical cancer education is needed to provide accurate information and to raise public awareness about cervical cancer screenings. Health providers should assume an active role in the provision of appropriate information about the importance of screenings to women’s health. This study draws our attention to the fact that the provision of knowledge about cervical cancer could reduce the anxiety and stigma associated with screenings and thus encourage participation. However, not all women who are willing to participate in screenings actually do participate in them. In-depth interviews are also warranted to determine how women’s willingness transfers to actual participation in screenings [29], [30].
